# The G protein-coupled receptor-associated protein 1 (GASP-1) regulates rimonabant-induced downregulation of GPR55

**DOI:** 10.1186/1471-2210-11-S2-A57

**Published:** 2011-09-05

**Authors:** Julia Kargl, Nariman A Balenga, Jennifer L Whistler, Maria Waldhoer

**Affiliations:** 1 Institute of Experimental and Clinical Pharmacology, Medical University of Graz, 8010 Graz, Austria; 2 Current address: Molecular and Signal Transduction Section, Laboratory of Allergic Diseases, NIAID/NIH, Bethesda, MD 20892-1889, USA; 3 Ernest Gallo Clinic and Research Center, University of California, San Francisco, CA 94608, USA; 4 Current address: Hagedorn Research Institute, Novo Nordisk A/S, 2820 Gentofte, Denmark

## Background

The G protein-coupled receptor 55 (GPR55) has recently been suggested to be responsible for those cannabinoid responses that could not be attributed to either the cannabinoid 1 (CB_1_) or cannabinoid 2 (CB_2_) receptor. Several potent GPR55 agonists were identified such as lysophosphatidylinositol (LPI) and several synthetic cannabinoids: One of these is rimonabant (SR141716A), an antagonist at the CB_1_ receptor, which showed clinical promise, but approval was revoked due to adverse events. Generally, the activity of G protein coupled receptors (GPCRs) is coordinated by receptor signalling, receptor desensitization and receptor resensitization. One regulatory mechanism to guarantee appropriate GPCR expression levels in physiological conditions is that of downregulating GPCRs via the G protein-coupled receptor-associated sorting protein 1 (GASP-1), thus leading to an attenuation of cellular signalling events. GASP-1 was originally found to target δ opioid receptors to lysosomes and, hence, to the degradative pathway. It was shown that GASP-1 is a key determinant in the development of analgesic tolerance to cannabinoids via its role in facilitating downregulation of the CB_1_ receptor.

## Methods and results

By a variety of approaches we demonstrated that rimonabant promotes downregulation of GPR55 via GASP-1 *in vitro* and *in vivo*. We show that GPR55 interacts with GASP-1 *in vitro* and that disrupting the GPR55/GASP-1 interaction prevents post-endocytic receptor degradation, and thereby allows receptor recycling. Together, these data implicate GASP-1 as an important regulator of rimonabant-mediated downregulation of GPR55.

## Conclusions

This work provides tangible evidence that GPR55 is degraded after prolonged agonist stimulation and that this mechanism is regulated by GASP-1.

